# The Evidence for Common Nonsurgical Modalities in Sports Medicine, Part 1: Kinesio Tape, Sports Massage Therapy, and Acupuncture

**DOI:** 10.5435/JAAOSGlobal-D-19-00104

**Published:** 2020-01-03

**Authors:** David P. Trofa, Kyle K. Obana, Carl L. Herndon, Manish S. Noticewala, Robert L. Parisien, Charles A. Popkin, Christopher S. Ahmad

**Affiliations:** From the Department of Orthopaedics, New York Presbyterian, Columbia University Medical Center, New York, NY (Dr. Trofa, Mr. Obana, Dr. Herndon, Dr. Noticewala, Dr. Popkin, and Dr. Ahmad); and the Department of Orthopaedics, Boston Medical Center, Boston University, Boston, MA (Dr. Parisien).

## Abstract

There are a number of nonsurgical modalities used by athletes in attempts to improve performance or prevent, treat, and rehabilitate musculoskeletal injuries. A concise review of available evidence on common nonsurgical modalities used today is necessary so that practitioners may appropriately counsel patients.

**Methods:**

A comprehensive review of relevant publications regarding Kinesio taping, sports massage therapy, and acupuncture from 2006 through 2019 was completed using PubMed and Google Scholar.

**Results:**

There have been numerous investigations evaluating the efficacy of nonsurgical modalities for a myriad of musculoskeletal conditions. There is some low level evidence to suggest the use of Kinesio tape for athletes with acute shoulder symptoms and acupuncture for carpal tunnel syndrome and as an adjunct treatment for low back pain. There is a need for higher quality research to better elucidate the effect of sports massage therapy on sports performance, recovery, and musculoskeletal conditions in general.

**Conclusions:**

Nonsurgical modalities are low-cost treatment strategies with very few reported adverse outcomes that will likely continue to increase in popularity. High-quality studies are needed to effectively evaluate these treatments, so that care providers can provide appropriate guidance based on evidence-based medicine.

The sports medicine specialist enjoys a unique role in the nonsurgical and surgical care of active patients of all skill levels. However, various nonsurgical modalities represent a notable element of sports medicine and musculoskeletal care that is not traditionally taught during orthopaedic training. The increasing use of such modalities merits a review of available evidence to appropriately assess their efficacy and safety, so that clinicians are able to make informed decisions in the care of patients requesting such therapy. In part 1 of this two-part series, we analyze Kinesio taping, sports massage therapy (SMT), and acupuncture. Part 2 will discuss cupping and blood flow restriction training.

## Kinesio Tape

### Background

Athletic taping is a common practice, thought to improve proprioception and muscular function by providing support and protection during active movement. Kinesio tape (KT) was developed in the 1970s by chiropractor Dr. Kenso Kase, and it has gained prominence in popular culture and athletics. Its use was especially bolstered during the 2008 Olympics when the tape was donated to 58 nations and worn by many high-profile athletes.^1,2^ According to the manufacturer, KT has specific properties that make it more effective than traditional athletic tape. From a functional standpoint, KT can stretch up to 140% to 160% of its original length and recoil back to apply a tensile force to the skin.^3^ It can also be worn longer and stick better to skin than traditional tape during athletic events because of its water resistance and breathability.^4^ Physiologically, KT purportedly improves proprioceptive signals to the brain and increases blood and lymphatic flow by lifting the skin off the underlying fascia. KT is also hypothesized to decrease pain by diminishing input from afferent nerve fibers and reducing pressure on subcutaneous nociceptors.^5,6^ Below we review the available evidence for the use of KT to investigate whether these claims provide a clinical benefit.

### Evidence

There are a limited number of high-quality clinical trials published evaluating KT. To the best of our knowledge, the first systematic review of KT was done in 2010 and included three randomized controlled trials (RCTs) comparing KT with sham taping for various musculoskeletal conditions.^7,8,9,10^ Among these, Thelen et al^7^ compared the short-term effects of KT and sham taping for rotator cuff tendinitis and impingement. The authors found that KT markedly improved pain-free shoulder range of motion (ROM) in abduction after immediate application, but not after 3 and 6 days. No differences in pain or disability scores were identified. Hsu et al^8^, in the only analysis of injured athletes, done a kinematic investigation in 17 baseball players with shoulder impingement comparing KT to athletic tape. KT increased the activity of the lower part of the trapezius muscle as measured by EMG immediately after taping. The clinical significance of this finding is unknown. Finally, Gonzalez-Iglesias et al^9^ investigated 41 patients with cervical neck pain and found a notable improvement in pain and ROM immediately after KT placement and after 24 hours.

Although the above three trials did identify some statistical significance results, perhaps pointing toward increasing the ROM of the shoulder and cervical spine and decreased pain within the first 24 hours of KT placement, the data have notable limitations. For instance, there was a lack of blinding for therapists providing the intervention and doing outcome measurements. More importantly, no trial showed any sustained benefit of taping given the short follow-up periods, and all statistically notable differences identified were so small that authors questioned their clinical significance. As such, this systematic review concluded that there was not sufficient evidence to support KT.^10^

In 2012, Williams et al^2^ published a second meta-analysis analyzing KT. Of included articles that investigated KTs role in increasing strength, Chang et al. showed no difference in maximal grip strength, while Hsu et al and Lee et al both showed an increase in maximal grip strength.^9,11,12^ Owing to these conflicting results, the meta-analysis could make no final recommendations regarding the use of KT for increasing grip strength. Regarding quadriceps strength, Vithoulk et al showed that KT in healthy women could increase quadriceps torque during eccentric exercise compared with no taping or sham taping, while Fu et al found that KT could improve peak torque during concentric quadriceps contractions.^13,14^ As such, Williams et al concluded that there is some evidence to suggest KT may a small benefit on patient strength. Among other findings, the authors concluded that there was no evidence to support KT use for ankle pain or proprioception, and that overall, further research was required to justify KT's use among a clinical population.^2,4^

KT has also been investigated as a treatment for chronic low back pain as reviewed in a meta-analysis of five studies by Nelson.^15^ Parreira Pdo et al^16^ compared a 4-week course of KT versus sham taping and found no differences in pain, disability, or global perceived effect at 12 weeks. Kachanathu et al. and Bae et al also showed no differences in pain or disability between cohorts either treated with KT in addition to traditional physical therapy versus physical therapy alone for 4 weeks or 12 weeks, respectively.^17,18^ Castro-Sánchez et al^19^ done 1 application of KT versus sham KT for a single week and reported a notable reduction in pain in their KT cohort at 1 and 4 weeks of follow-up. And, finally, Paoloni et al^20^ showed no difference in pain or disability scores after 4 weeks of treatment with either KT alone, KT plus therapeutic exercise, or exercise alone. Nelson agrees with previous KT meta-analyses in that KT may provide a transient, although perhaps not clinically significant, reduction in pain, and perhaps an improvement in endurance after 4 weeks of KT, but once again points out the lack of high-quality evidence to make a strong recommendation for its use. Finally, in an RCT, Macedo et al^21^ reported KT applied with and without tension markedly decreased low back pain at 3 days, but not 10 days, after application compared with those without KT. Macedo et al also found a notable reduction in disability through 10 days in patients treated with KT compared with those without. These findings support the meta-analysis by Nelson^15^ in which the effectiveness of KT in reducing long-term low back pain is questionable.

KT has also been studied for the treatment of osteoarthritis (OA) in two RCTs comparing KT with sham KT. Cho et al^22^ evaluated the immediate effects of KT within 1 hour of application on elderly patients with radiographically confirmed OA. The authors found that compared with the sham cohort, patients receiving KT experienced notable improvements in pain, ROM, and proprioception of the affected knee. Kocyigit et al^23^ compared patients who wore KT and sham KT continuously for 12 days. The authors found that both KT and sham KT improved pain, and that both groups had an increase in functional performance, but that real KT was not superior to sham KT. Similarly, Wageck et al^24^ evaluated knee concentric muscle strength and pain in adults with OA treated with KT or sham tape. The authors did not find an improvement or difference in knee strength or pain through the 19-day follow-up period. It should also be noted that the above studies did not differentiate the severity of OA being treated radiographically by the Kellen Lawrence scale.

### Conclusion

Although KT has been recently popularized and is widely used, there is a lack of convincing and well-designed evidence to support its use for musculoskeletal conditions. There may be a small immediate effect of KT on pain and ROM in some joints, but further high-quality studies are needed to rationalize its use for patients. There is some evidence that suggests it may be reasonable to apply KT in the athletic patient with shoulder symptoms in the immediate preactivity/sport setting. Given that no studies have ever reported an adverse outcome associated with KT, its safety, affordability, and popularity may continue to make in an attractive option to patients.

## Sports Massage Therapy

### Background

Therapeutic massage is the practice of manipulating muscles and limbs to ease tension and reduce pain. Massage therapy dates back thousands of years, with the first written records linked to ancient Chinese and Egyptian culture where oils and herbs were used as an adjuvant to address muscle pain. The practice of therapeutic sports massage developed alongside athletics in ancient China where it was used by Taoist priests for the treatment of practitioners of Kung Fu, as well as in ancient Greece in the care of Greek Olympians. Today, massage is widely practiced and taught in Chinese hospitals and medical schools and originally gained popularity in the United States in the 19th century on its introduction by two New York physicians.^25^ The practice of SMT was first offered as a core medical service to the US Olympic team at the 1996 Summer Olympic Games in Atlanta and is now regularly used by many professional, Olympic, and collegiate athletes to address the varying physical sequelae of athletic competition.^26^

There is a myriad of types and styles of massage that use several pieces of fundamental equipment depending on the particular technique. Specialized padded massage tables and ergonomic chairs allow for appropriate positioning while U-shaped head supports enable an ease of breathing as the athlete lies prone. Low-cost, easily accessible equipment, such as foam rollers, is also used for deep muscle massages before and after workout.

### Evidence

SMT has been proposed as a means to help prepare athletes for competition through enhancement of athletic performance, as a treatment approach to aid in recovery after competition, and as a direct intervention for sports-related musculoskeletal injuries.^27,28^ Physical therapists, athletic trainers, and certified massage therapists are the practitioners commonly doing SMT in the athletic arena with limited supporting evidence. A comprehensive meta-analysis of massage therapy research conducted by researchers at the University of Illinois at Urbana-Champaign evaluated several variables and factors from 37 studies which included gate control theory of pain reduction, promotion of parasympathetic activity, influence of body chemistry, mechanical effects, promotion of restorative sleep, and interpersonal attention. The authors concluded that the current body of evidence “supports the general conclusion that massage therapy is effective.”^29^

Proper flexibility and strength are vital factors with regard to injury prevention and overall performance in competitive athletes. Studies on the effect of SMT on flexibility have demonstrated positive short-term outcomes with multiple studies evaluating hamstring flexibility in physically active young men and female field hockey players receiving a classic massage and deep tissue massage.^30–32^ Strength is another key component to the overall health and success of high-level athletes. The assessment of therapeutic massage on power grip strength in a pre-test and post-test study design by Brooks et al^33^ reported superiority in postexercise grip performance in those patients having undergone massage intervention. Therefore, the authors conclude that applying massage for 5 minutes shortly after fatiguing exercises is beneficial. An additional investigation by Mancinelli et al^34^ evaluated the effects of SMT on female collegiate athletes done at the beginning of the basketball and volleyball seasons. The study consisted of 22 National Collegiate Athletic Association Division-I women's basketball or volleyball players who underwent a 17-minute massage. The authors reported that the massage intervention markedly increased the athlete's vertical jump and decreased their perceived soreness. Moran et al.^35^ conducted a randomized, counterbalanced, repeated measures experiment to investigate the effect of precompetition massage compared with a traditional warm-up, massage and warm-up, and ultrasonography control on 60-meter sprint times in 17 NCAA track and field athletes. The authors found no notable differences between any of the interventions with respect to sprinter speed or acceleration. Macgregor et al^36^ conducted a randomized, counterbalanced, repeated-measures experiment to assess muscular efficiency and flexibility in 16 males after foam rolling compared with no intervention controls. The authors found that maximal voluntary contraction of muscles was markedly greater in patients using the foam roll for three days compared with the control group, although range of motion was not markedly different. Conversely, Hodgson et al^37^ conducted an RCT comparing ROM and jump performance of active students who foam rolled three times a week, six times a week, or not at all. The authors found no consistent training-induced changes from foam rolling.

In attempt to examine specific pathologies, a randomized single-blinded study evaluating the therapeutic effects of massage on pain, muscle tension, and anxiety in patients with scapulothoracic pathology reported statistically notable improvement in pain intensity and muscle tension.^38^ However, overall shoulder function was not analyzed. An additional meta-analysis consisting of 12 RCTs evaluating the impact of massage therapy on neck and shoulder pain suggests immediate positive effects of massage therapy on both shoulder and neck pain with short-term positive effects for shoulder pain only. However, functional status of the shoulder was not found to be markedly affected by massage therapy.^39^

Associated pathology and disorders of the knee are also common findings in athletes for which friction massage and several nonsurgical modalities have been compared. Stasinopoulos et al evaluated the effectiveness of transverse friction massage compared with a focused exercise program and pulsed ultrasonography in a cohort of patients in their 20 and 30 seconds with patellar tendinopathy.^40^ The authors found an exercise program to be more effective than both pulsed ultrasonography and transverse friction massage immediately after treatment and at 3 months of follow-up.^40^ In further evaluation of the effect of therapeutic massage on musculoskeletal disorders, Bervoets et al^41^ done a systematic review of 26 studies consisting of 2,565 patients. The authors concluded that therapeutic massage reduced pain and improved function compared with no treatment for some musculoskeletal conditions. In addition, a comprehensive review of the literature broadly examining the effect of massage on sports performance was conducted with the inclusion of studies investigating the use of massage in all facets of athletic care and concluded that “poor appreciation exists for the appropriate clinical use of sports massage” with additional studies required to further examine the “physiological and psychological effects of sports massage.”^42^

### Conclusion

Despite multiple independent studies and subsequent meta-analyses, there is a strong need for higher quality research examining the efficacy and acceptability of massage therapies in the athletic patient population to determine a more direct correlation between the effects of SMT on sports performance and recovery, as well musculoskeletal conditions in general.

## Acupuncture

### Background

Acupuncture is a form of complementary and alternative medicine used by many patients to treat a variety of disorders. Acupuncture originated in China over 2,000 years ago, and its popularity in the West has increased.^43^ A recent survey by the National Institute of Health in the United States found that more than three million Americans had undergone treatment with acupuncture at least once in the previous year.^44^ Traditional acupuncture involves a specific pattern of placing needles at certain locations on the body, called meridians. In traditional teachings, the goal of acupuncture is to balance yin and yang forces within the body, yin being the cold and slow force and yang the hot and excited. A blockage of the flow of these energies, or qi, was believed to be a source of pain and pathology. Therefore, it was believed that placing needles along meridians of the body would unblock that flow and restore balance. In a more Western physiological sense, it is believed that acupuncture works by stimulating trigger points in various muscle groups promoting the release of endogenous hormones, such as endomorphin, encephalin, and serotonin, and creating an analgesic effect.^45^ Although the exact mechanism by which acupuncture exerts its effects continues to be debated, its practice has been evaluated clinically for many different musculoskeletal applications.

### Evidence

For chronic low back pain, there are several recent systematic reviews and meta-analyses investigating the efficacy of acupuncture. For instance, a 2005 Cochrane review included 22 RCTs evaluating acupuncture.^46^ The authors concluded that compared with no treatment, acupuncture can provide pain relief and functional improvement at short-term follow-up. When compared with sham acupuncture, acupuncture showed some pain relief in short term but not long-term follow-up and no improvement in function. When compared with other alternative treatments (such as massage), acupuncture showed no improvement in pain or function, but when added to conventional treatments (such as exercise programs or physical therapy), acupuncture improved pain scores and functional outcomes compared with conventional therapies alone. Other reviews are less enthusiastic, stating there is no evidence to suggest that acupuncture provides analgesia for the neck or back compared with placebo or sham treatment,^47–49^ while others claim it may be better than placebo, but no better than other treatment modalities such as spinal manipulation or massage.^50^

The most current systematic review of the literature was done by Ammendolia et al^43^ in 2008. The authors reviewed 19 RCTs and concluded that acupuncture was not superior to any treatment for immediate and short-term pain relief, or immediate functional improvement, but was superior to no treatment for short-term functional outcomes. However, there was good evidence that acupuncture, when added as an adjunct to other treatments such as NSAIDs, non-narcotic analgesics, mud packs, infrared heat therapy, back care education, ergonomics, and behavioral modification, improved patient pain symptoms and functional outcomes compared with those interventions alone.

In light of the available data, several large bodies have recently released recommendations regarding the use of acupuncture for back pain. The European guidelines do not recommend acupuncture for the treatment of chronic, nonspecific low back pain.^51^ In the United States, the American College of Physicians and the American Pain Society recommend that for patients who have low back pain that is refractory to conventional medical care and exercise, adjunct treatments may be used. Acupuncture has a “weak recommendation” because of the availability of only moderate-quality evidence.^52^

Acupuncture has also been described for treatment of musculoskeletal problems of the upper and lower extremity. In a recent review by Cox et al,^53^ 15 RCTs were reviewed for various ailments of the extremity. For carpal tunnel syndrome, it was found that there was a notable improvement in patient-reported symptoms such as pain and numbness with traditional acupuncture when compared with patients receiving prednisolone injections, sham acupuncture, night splinting, and vitamin B supplements.^54–56^ The authors point out that these statistically notable results may lack clinical significance because the differences were small. Acupuncture showed no improvement in pain in patients with recent-onset plantar fasciitis compared with placebo.^57^ Finally, there was no difference in patient-reported pain at 5 months in patients with patellofemoral pain syndrome treated with acupuncture compared with no treatment.^58^

Systematic reviews have evaluated acupuncture for treatment of various ailments of the shoulder.^50,59^ Green et al^59^ showed that acupuncture did no better than placebo in relieving pain or improving ROM in patients with rotator cuff disease. There is evidence that acupuncture, when added to exercise is more effective in increasing ROM and decreasing pain in patients with adhesive capsulitis when compared with acupuncture alone.^60^ Acupuncture was equivalent to ultrasonography treatment for pain relief in patients with impingement.^61^ For nonspecific shoulder pain, acupuncture showed statistically, but not clinically notable reduction in pain after 3 months of treatment.^62^ Green et al^59^ concluded that there is evidence that acupuncture can provide some short-term relief for specific shoulder pathologies (such as adhesive capsulitis), while more evidence is needed for a stronger recommendation regarding its use. On the other hand, Cox et al^53^ concluded that the evidence for acupuncture in patients with shoulder pathology is inconclusive.

Acupuncture has also been investigated for other upper extremity pathologies, including lateral elbow pain, lateral epicondylitis, and bicep brachii pain.^53,63,64^ Gadau et al reviewed 19 RCTs concluding that acupuncture was more effective than sham acupuncture. However, they note that every study included had at least one flaw in terms of bias, and that the longest length of follow-up for any study was 6 months. They are thus cautious in their final conclusion, pointing out the need for further high-quality evidence to make a more informed conclusion.^63^ Cox et al^53^ done a sensitivity analysis of several highly flawed trials claiming that acupuncture provided benefit for the treatment of lateral epicondylitis, but concluded that any evidence of benefit was likely due to methodological bias and therefore not useful in making recommendations. Finally, Mansfield et al conducted a systematic review and meta-analysis of 21 studies analyzing muscle force production. The authors found no benefit of acupuncture in muscle strength or pain of the shoulder after acupuncture compared with physical therapy.^64^ Based on the available literature, no recommendation can therefore be made regarding the use of acupuncture to treat upper extremity pathology.

A new area of research for acupuncture is as an ergogenic aid in sports performance. Several studies have been done on both athletes and nonathletes to assess whether acupuncture can increase performance. Ozerkan et al^65^ showed an increase in knee flexion and extension isokinetic strength in young soccer players after an acupuncture regimen compared with their baseline before acupuncture. Other studies have failed to show a performance benefit of acupuncture, demonstrating no benefit in one-legged vertical jump when compared with placebo.^66^ For endurance athletes, acupuncture has been shown to be equivalent to both placebo and sham acupuncture in nonathletes in terms of heart rate, rating of perceived effort, oxygen uptake, and ventilation at submaximal effort during a cycling exercise and showed an insignificant time difference in a 20-km cycling test in experienced cyclists when compared with no treatment and sham acupuncture.^67^ However, these studies only looked at immediate results of acupuncture, and no long-term follow-up was included. There are limited data regarding acupuncture as a performance aid, and the available literature is conflicting regarding whether acupuncture does provide any performance advantage.

In terms of safety, only a minority of studies included reports on adverse events. Reported adverse events included ecchymosis, transient paresthesia, fainting, and dizziness.^53^

### Conclusion

There is insufficient evidence to definitively conclude that acupuncture is an effective treatment modality for musculoskeletal disorders. Some studies have shown a benefit of acupuncture, particularly in the immediate relief of pain for carpal tunnel syndrome and as an adjunct for the treatment of low back pain. Meanwhile, the data evaluating acupuncture as a tool to treat various pathologies of the shoulder and elbow are inconclusive, as is the data relating to the use of acupuncture as a performance enhancing modality. Given the popularity, safety, relatively low cost, anecdotal evidence, and potential of this nonsurgical modality, acupuncture deserves to be investigated through high-quality, unbiased randomized controlled trials with procedure standardization.
